# Targeted Disruption of *LDLR* Causes Hypercholesterolemia and Atherosclerosis in Yucatan Miniature Pigs

**DOI:** 10.1371/journal.pone.0093457

**Published:** 2014-04-01

**Authors:** Bryan T. Davis, Xiao-Jun Wang, Judy A. Rohret, Jason T. Struzynski, Elizabeth P. Merricks, Dwight A. Bellinger, Frank A. Rohret, Timothy C. Nichols, Christopher S. Rogers

**Affiliations:** 1 Exemplar Genetics, Coralville, Iowa, United States of America; 2 Departments of Medicine and Pathology and Laboratory Medicine, University of North Carolina, Chapel Hill, North Carolina, United States of America; Northeast Ohio Medical University, United States of America

## Abstract

Recent progress in engineering the genomes of large animals has spurred increased interest in developing better animal models for diseases where current options are inadequate. Here, we report the creation of Yucatan miniature pigs with targeted disruptions of the low-density lipoprotein receptor (*LDLR*) gene in an effort to provide an improved large animal model of familial hypercholesterolemia and atherosclerosis. Yucatan miniature pigs are well established as translational research models because of similarities to humans in physiology, anatomy, genetics, and size. Using recombinant adeno-associated virus-mediated gene targeting and somatic cell nuclear transfer, male and female *LDLR+/−* pigs were generated. Subsequent breeding of heterozygotes produced *LDLR−/−* pigs. When fed a standard swine diet (low fat, no cholesterol), *LDLR+/−* pigs exhibited a moderate, but consistent increase in total and LDL cholesterol, while *LDLR−/−* pigs had considerably elevated levels. This severe hypercholesterolemia in homozygote animals resulted in atherosclerotic lesions in the coronary arteries and abdominal aorta that resemble human atherosclerosis. These phenotypes were more severe and developed over a shorter time when fed a diet containing natural sources of fat and cholesterol. *LDLR*-targeted Yucatan miniature pigs offer several advantages over existing large animal models including size, consistency, availability, and versatility. This new model of cardiovascular disease could be an important resource for developing and testing novel detection and treatment strategies for coronary and aortic atherosclerosis and its complications.

## Introduction

The application of gene targeting technology in livestock animals, particularly pigs, has generated significant interest in the development of new models of human diseases. In cases where existing rodent models fail to recreate key clinical phenotypes, pigs are an attractive alternative. Perhaps the best example of this is the recent development of porcine models of cystic fibrosis (CF) [1], [2]. Pigs expressing either null alleles or the common F508del mutation in the cystic fibrosis transmembrane conductance regulator (*CFTR*) gene closely recapitulate human CF, including lung disease [3], [4]. These models have been important in elucidating new pathogenic mechanisms and uncovering previously unrecognized consequences of the disease; advances that were not possible in prior models of CF [5], [6]. Pigs also offer an advantage when modeling diseases for which size is relevant to the development of interventional devices and diagnostic equipment for humans, such as atherosclerosis [7].

Atherosclerosis is characterized by the accumulation of proliferative smooth muscle cells, macrophages, lipids, cholesterol, calcium deposits, and cellular debris in vessel walls [8]–[10]. Atherosclerosis begins with the buildup of low-density lipoprotein (LDL) in the subendothelial matrix [11]. This accumulation is proportional to the levels of circulating LDL. These processes result in plaque formation, arterial obstruction, and diminished blood flow to organs. For unknown reasons, these plaques can rupture, often when quite small, and lead to occlusive thrombosis, resulting in myocardial infarction or stroke [12]–[14].

In humans, loss-of-function mutations in the LDL receptor (*LDLR)* gene lead to familial hypercholesterolemia (FH) [15]. As its name implies, FH is associated with elevated plasma concentrations of total and LDL cholesterol. Approximately one in 500 people carries a mutation in one *LDLR* gene. These heterozygotes typically have twice the normal plasma LDL levels and a significantly increased risk for atherosclerosis. Individuals with two defective *LDLR* alleles face early-onset atherosclerosis and often die of the disease at a young age.

Several small and large animal models of hypercholesterolemia and atherosclerosis are available today, but each has limited utility. Murine models of atherosclerosis have been developed using transgenic and gene targeting techniques [16]–[19]. While these models have provided valuable insight into our current understanding of cardiovascular disease, differences between murine atherosclerosis and the corresponding human disease have hampered translation to the clinic [20]. For example, atherosclerosis in mice develops above the aortic valve and progresses in an antegrade fashion. Abdominal aortic atherosclerosis in humans begins at the aortoiliac junction and progresses in a retrograde fashion [21]. Also, mice typically do not develop coronary artery atherosclerosis except in the most proximal regions, and these lesions appear to be a direct extension of supra-aortic valve atherosclerosis as opposed to developing separately in the coronary arteries. In contrast, humans develop disease throughout the coronary artery system. Another key limitation of the murine models is size - the small size of the mouse limits their use in the development of human devices (such as stents) and new imaging technologies. Rabbit models of atherosclerosis have also been studied extensively [22]. While they do present a more accurate model of human atherosclerosis, like mice, their smaller size limits their broad application.

Pigs have long been studied as models of human cardiovascular disease, primarily due to similarities of their cardiovascular systems and their more human-like size [23]. For this reason, the pig is a preferred animal for testing in the cardiovascular device industry [24], [25]. Spontaneous atherosclerosis in pigs is rare, but when fed a diet high in saturated fat and cholesterol, pigs can develop advanced atherosclerotic lesions similar in type and location to those seen in humans [26]–[29]. However, these diets are incredibly expensive and require a long time to manifest disease, making many studies cost-prohibitive.

Pigs with spontaneous hypercholesterolemia and FH have been described by Rapacz and others [30]–[32]. These pigs harbor a point mutation (R84C) in both *LDLR* alleles that reduces receptor binding, as well as allele variations in *ApoB* that may further contribute to the phenotype. Even when consuming a normal diet, these pigs develop hypercholesterolemia and atherosclerotic lesions ranging from fatty streaks to advanced plaques, with accompanying calcification, neovascularization, hemorrhage, and rupture [33]. Despite the promising phenotype, there are significant drawbacks to the Rapacz FH pig. First, there is substantial variability in the plasma cholesterol levels and disease development [32]. This is likely due to the mild nature of the mutation and the broad genetic background of these animals. Second, the mutations were originally described in a large domestic pig breed, with body weights in excess of 200 kg. Efforts to downsize the pig by crossing with various miniature pig breeds has resulted in smaller pigs, but has also led to greater phenotypic variability [34].

Recently, the development of transgenic Yucatan miniature pigs expressing a proprotein convertase subtilisin/kexin type 9 (*PCSK9*) gain-of-function mutation (D374Y), which is associated with human autosomal dominant FH, was reported [35]. These pigs exhibit moderate hypercholesterolemia on standard pig feed, and a more severe hypercholesterolemia and human-like atherosclerosis following extended feeding of a high-fat, high cholesterol diet. However, with the mutant transgene being permanently overexpressed at nearly 500 times the normal level (hepatic mRNA expression), these pigs may have limited utility for treatments designed to increase LDL receptor expression or reduce PCSK9 activity.

We endeavored to address the disadvantages of the current models while maintaining the positive attributes of pigs in accurately modeling human cardiovascular disease. We used gene targeting and somatic cell nuclear transfer (SCNT) to create Yucatan miniature pigs with targeted disruptions in one or both *LDLR* alleles. We also evaluated the extent of hypercholesterolemia and atherosclerosis with both standard and high fat, high cholesterol diets.

## Methods

### Ethics Statement

This study was carried out in accordance with the recommendations in the Guide for the Care and Use of Laboratory Animals of the National Institutes of Health. The Institutional Animal Care and Use Committee of Exemplar Genetics approved all animal experiments. All animals were housed in Exemplar Genetics' AAALAC-accredited facilities. Standard procedures for animal husbandry were used throughout.

### Fetal Fibroblast Isolation

Fetal fibroblasts were previously isolated from approximately day 35 Yucatan miniature pig fetuses as previously described [36]. Fetus genders were identified by PCR amplification of the Y-chromosome-specific *Sry* gene [37].

### Cloning porcine LDLR genomic DNA

Genomic DNA was isolated from Yucatan fetal fibroblasts (Qiagen). A 8.6 kb PCR product which included a region from LDLR exon 2 to exon 6 was amplified using a high fidelity polymerase (Platinum Taq High Fidelity; Invitrogen) and LDLR primers 2F2 and 6R2 (see Table S1 for primer sequences). The PCR product was subcloned into pCR2.1-TOPO (Invitrogen) and sequenced. All DNA sequencing was performed by the University of Iowa DNA Facility. This plasmid (referred to as pLDLR2/6) served as the template for PCR amplification of the 5′ and 3′ homologous targeting arms.

### Targeting Vector Construction

The 5′ and 3′ homology arms were amplified by PCR using plasmid pLDLR2/6 and subcloned sequentially into a plasmid containing a PGK-Neo^R^ cassette. The primers for the 5′ arm were LDLR5′armF (*Xho*I) and LDLR5′armR (*EcoR*V). The primers for the 3′ arm were LDLR3′armF (*Hind*III) and LDLR3′armR (*Hind*III). The engineered termination codon is at amino acid position C128 (C128X). This targeting construct (pLDLR-Neo) was used as a template to create the amplicon for the generation of the *LDLR*-targeting proviral vector.

### rAAV production

PCR amplification of a 4.5 kb amplicon from plasmid pLDLR-Neo was achieved by using the following primers: AAV-LDLR-F (*Not*I) and AAV-LDLR-R (*Not*I). This product was subcloned into the rAAV proviral plasmid, pFBAAV2-CMVP.NpA (obtained from University of Iowa Gene Transfer Vector Core) and grown in Sure2 cells (Stratagene) to ensure ITR integrity. This proviral plasmid is referred to as pAAV-LDLR-Neo. The rAAV targeting virus was produced by the University of Iowa Gene Transfer Vector Core.

### Fetal fibroblast infection, selection, and screening

rAAV infection, antibiotic selection, and PCR screening (using primers LDLR Ex4aF1 and LDLR Int4R1) were performed as previously described [1]. The expected PCR screening product for the wild-type allele was 1.5-kb and 3.2-kb product from the *LDLR*-targeted allele.

### Southern blot

Genomic DNA isolation, whole genome amplification, and Southern blotting procedures were performed as previously described [1]. The DNA probes for *LDLR* and *Neo^R^* were produced by PCR amplification using the following primers: LDLR *Xmn*I probe1F/LDLR *Xmn*I probe 1R and PGK-NeoF/NeoR-R, respectively.

### Production of pigs by SCNT

Nuclear transfer was performed by Viagen, Inc. (Austin, TX) as previously described [38]. Reconstructed oocytes were transferred into synchronized post-pubertal gilts on the first day of standing estrus. Following a midline incision to access the uterus, reconstructed embryos were transferred into the oviduct at the ampullary-isthmus junction. Recipient animals were checked for pregnancy by abdominal ultrasound after day 21 and throughout gestation.

### Northern blot

Total RNA was isolated (RNaqueous-4 PCR; Ambion) from *LDLR+/+*, *LDLR+/−*, and *LDLR−/−* pig liver. Poly A^+^ RNA was obtained from liver total RNA by using FastTrack MAG mRNA Isolation Kits (Invitrogen). Northern blot analysis was performed using the NorthernMax formaldehyde-based system (Ambion). *LDLR* and *GAPDH* probe templates were amplified using the primer sets LDLR North8F/9R and pGAPDH North1F/2R, respectively.

### Reverse Transcription PCR

Total RNA was isolated as described above. First strand cDNA was synthesized with 2 μg of total RNA using SuperScript III (Invitrogen) and following manufacturer's instructions. PCR was performed using primers LDLR North4F and LDLR Exon 5R1, which spans *LDLR* exons 1–5.

### Western blot

Pig liver was homogenized on ice in RIPA buffer (1% NP-40, 1% Na-deoxycholate, 0.1% SDS, 0.15 M NaCl, 0.01 M NaPO4 pH 8.0, 2 mM EDTA) supplemented with protease inhibitor cocktail (Sigma), 1 mM PMSF (Sigma), and 1 mM DTT. The homogenate was centrifuged at 4°C, 10,000×g for 30 minutes, and the supernatant was combined with SDS-PAGE sample buffer (50 mM Tris-HCl pH 6.8, 2% SDS, 10% glycerol, 100 mM DTT, 0.1% bromophenol blue). The liver lysate samples were heated in boiling water for 5 minutes, cooled on ice, and sonicated with a Sonic Dismembrator (Fisher Scientific). The samples were electrophoresed in a 7.5% SDS-PAGE gel with Tris-Glycine running buffer. Protein was transferred onto a PVDF Immobilon-P Transfer Membrane (Millipore) using XCell II Blot Module (Invitrogen) according to manufacturer's instructions. Immunoblot analysis was performed using primary antibodies against LDLR (EP1553Y, Abcam), β–tubulin (SC-55529, Santa Cruz Biotechnology), and secondary antibodies conjugated with horseradish peroxidase (SC-2317 and SC-2005, Santa Cruz Biotechnology). Signal was detected using Chemiluminescent Detection Kit (Pierce).

### Lipid chemistry

Whole blood was collected in EDTA-containing tubes, and plasma was prepared and frozen at −80°C. Lipid analysis of most samples was performed by the Lipoprotein Analysis Laboratory in the Department of Pathology at Wake Forest University School of Medicine (Winston-Salem, NC). Total cholesterol was measured by enzymatic assay, lipoproteins were measured by gel filtration chromatography. Total cholesterol of samples from 15–18 month old heterozygote animals was determined by LipoScience (Raleigh, NC).

### Atherosclerosis measurements and morphometric analysis

The heart and coronary arteries from female *LDLR+/+* and *LDLR−/−* littermates were flushed with normal saline and perfusion-fixed under systemic pressure with 10% buffered formalin. Abdominal aortas were immersion-fixed in 10% buffered formalin. Sections of each coronary artery were taken perpendicular to the direction of blood flow at one cm intervals from their origin and embedded in paraffin. The number of sections reflects the distance covered. In general there are an average of 20 total coronary artery sections per pig heart: 7 left anterior descending coronary artery, 10 right coronary artery, 3 to 4 circumflex. The coronary artery sections were stained with Verhoeff-van Gieson to highlight the internal and external elastic lamina. Adjacent sections were stained with hematoxylin and eosin to confirm lesion details. The abdominal aorta between the renal and iliac arteries was photographed for measuring the percent surface area with raised lesions when macroscopically present as follows: % surface area with raised lesions  =  (area with raised lesions ÷ total surface area) ×100. Measurements were confirmed by Sudan IV staining. The abdominal aorta was then divided into 1 cm sized opened segments taken perpendicular to the direction of blood flow from the level of the renal arteries to the origin of the iliac arteries, producing on average 6 sections per pig and paraffin embedded. Light microscopic sections were prepared from the proximal half of each segment and stained with Verhoeff-van Gieson and hematoxylin and eosin.

### Statistics

Analysis for significance was performed with GraphPad Prism software (GraphPad Software) using one-way ANOVA with post hoc comparison using the Tukey test or unpaired Student's t test, as specified.

## Results

### Targeted disruption of porcine *LDLR*

**Figure 1:**
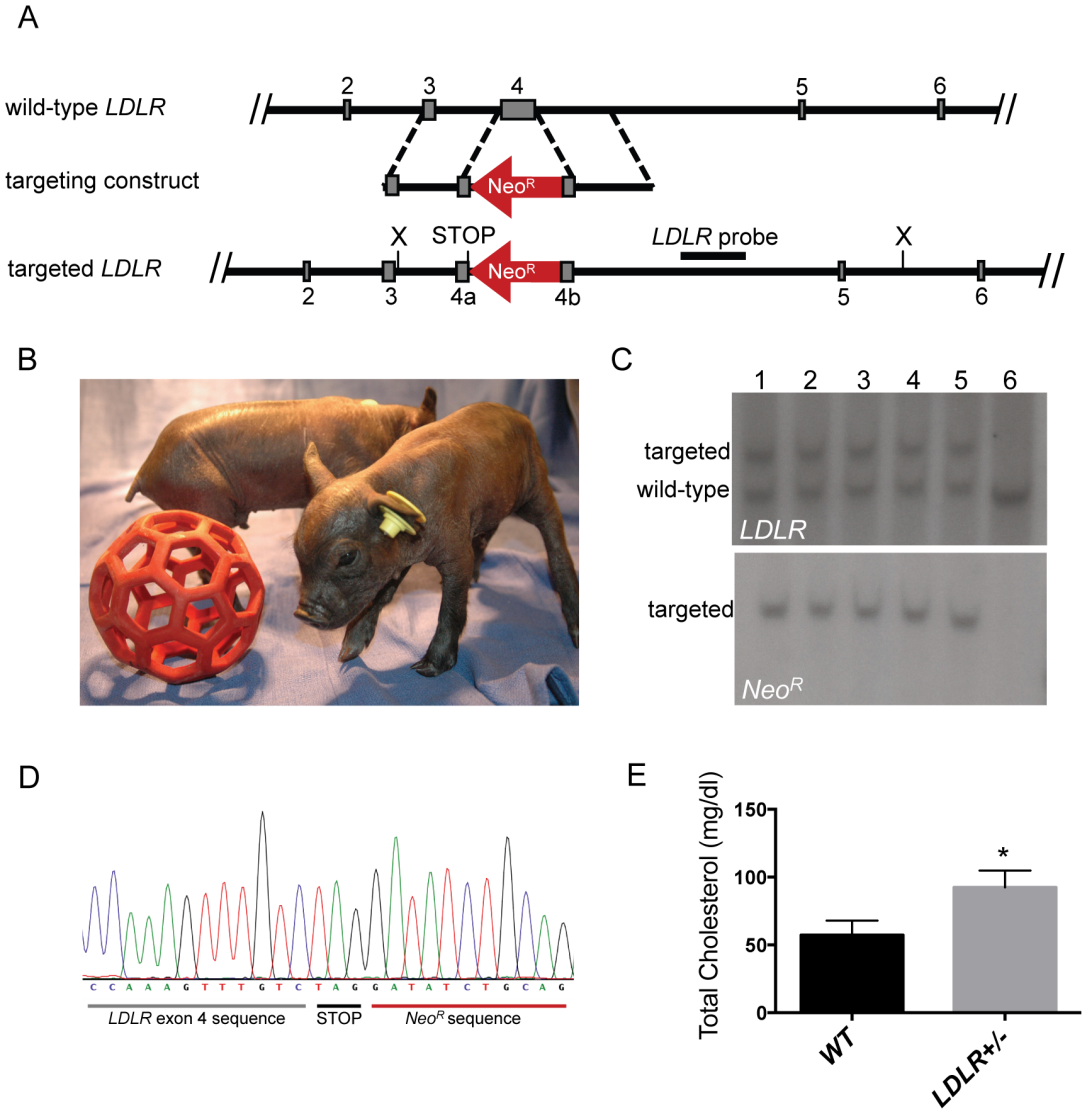
*LDLR*-targeted pigs. A. Gene targeting strategy. Wild-type *LDLR* exons 2–6 are shown as gray boxes. *Neo^R^* cassette is depicted in red, with the arrow showing the orientation relative to *LDLR*. The neomycin resistance cDNA is driven by a *PGK* promoter and flanked by *loxP* sites. The engineered termination codon is indicated. Position of the *LDLR* Southern blot probe is shown, as are the *Xmn*I restriction sites. B. *LDLR+/−* piglets at 2 days of age. C. Southern blot of genomic DNA from *LDLR+/−* cloned pigs. (Upper) *XmnI* digested genomic DNA was hybridized with a probe that detects porcine *LDLR* downstream of the targeting vector boundary. The *LDLR*-targeted allele produced an approximately 7.8 kb band, and the wild-type band is approximately 6.0 kb. (Lower) The same DNA was hybridized with a probe that detects the *Neo^R^* cassette, yielding only the targeted 7.8 kb band. Lanes 1–5 contain DNA from individual cloned *LDLR+/−* pigs; lane 6 contains DNA from a wild-type pig. D. Sequence chromatogram of the site of *LDLR* disruption by the *Neo^R^* cassette. The engineered termination codon is noted. E. Total cholesterol levels in plasma from 15–18 month old *LDLR+/−* pigs (n = 13) and wild-type controls (n = 7), *p<0.0001. Data presented with SD.

Homologous recombination was used to disrupt the porcine *LDLR* gene. Specifically, a neomycin-resistance cassette (*Neo^R^*) was inserted into exon 4 of porcine *LDLR* (Figure 1A). Exon 4 encodes a necessary portion of the LDL receptor's ligand-binding domain [39]. It is a frequent site of mutation in human FH and was also targeted in a murine model, resulting in the loss of LDL receptor function [17]. Additionally, a premature termination codon was engineered immediately upstream of the *Neo^R^* insertion to ensure that no functional protein was produced.

Recombinant adeno-associated virus (rAAV) was used to deliver the targeting vector to male and female fibroblasts harvested from Yucatan miniature pig fetuses. Following rAAV infection and antibiotic selection, *LDLR*-targeted fibroblasts were identified using PCR (Figure S1A). The gene targeting efficiency (1.8% in male cells and 6.5% in female cells) was comparable to other studies using rAAV to target porcine genes (Table 1) [1], [40]. Several PCR-positive cell lines were further confirmed by Southern blot analysis to contain the expected *LDLR* targeting event and were free of random integration (Figure S1B).

**Table 1:** Summary of *LDLR* gene targeting and SCNT activity.

	Gene targeting efficiency*	Number of transfers	Embryos per transfer (average)	Pregnancy rate†	Live pigs per litter	Total Live Born
**Male**	1.8%	10	130	50%	4.8	24
**Female**	6.5%	8	122	50%	8.3	33

*Gene targeting efficiency reported as percentage of G418^R^ clones that were properly targeted, as determined by PCR.

† Pregnancy rate refers to full-term gestation.

### Generation of *LDLR+/−* pigs by SCNT

*LDLR+/−* cells were used as nuclear donors for SCNT [38]. We performed 18 embryo transfers yielding 9 full term pregnancies and 57 *LDLR+/−* piglets (24 male and 33 female) (Figure 1B). Southern blot and DNA sequencing confirmed the expected genotypes (Figure 1C,D).

Naturally occurring FH pigs have previously been shown to inherit the disease in a recessive manner with heterozygotes displaying no significant elevation in total cholesterol [32]. To assess whether this was true in *LDLR*-targeted Yucatan miniature pigs, total cholesterol was measured in a group of 15 to 18 month old male *LDLR+/−* cloned pigs (n = 16) and wild-type controls (n = 7) fed a standard diet (∼2% fat and no cholesterol). *LDLR+/−* pigs total cholesterol was elevated relative to control pigs (*LDLR+/+*: 57.3±4.0 and *LDLR+/−*: 92.4±5.6, p<0.0001) (Figure 1E).

### Production of *LDLR−/−* pigs by breeding

Cloned *LDLR+/−* males and females were retained for breeding purposes. For this study, sixteen litters were produced and yielded the expected Mendelian inheritance of 20 *LDLR+/+*, 40 *LDLR+/−*, and 21 *LDLR−/−* pigs. Since the sires and dams were each derived via SCNT from single, *LDLR*-targeting events, each litter produced from these matings is essentially from the same cross.

**Figure 2:**
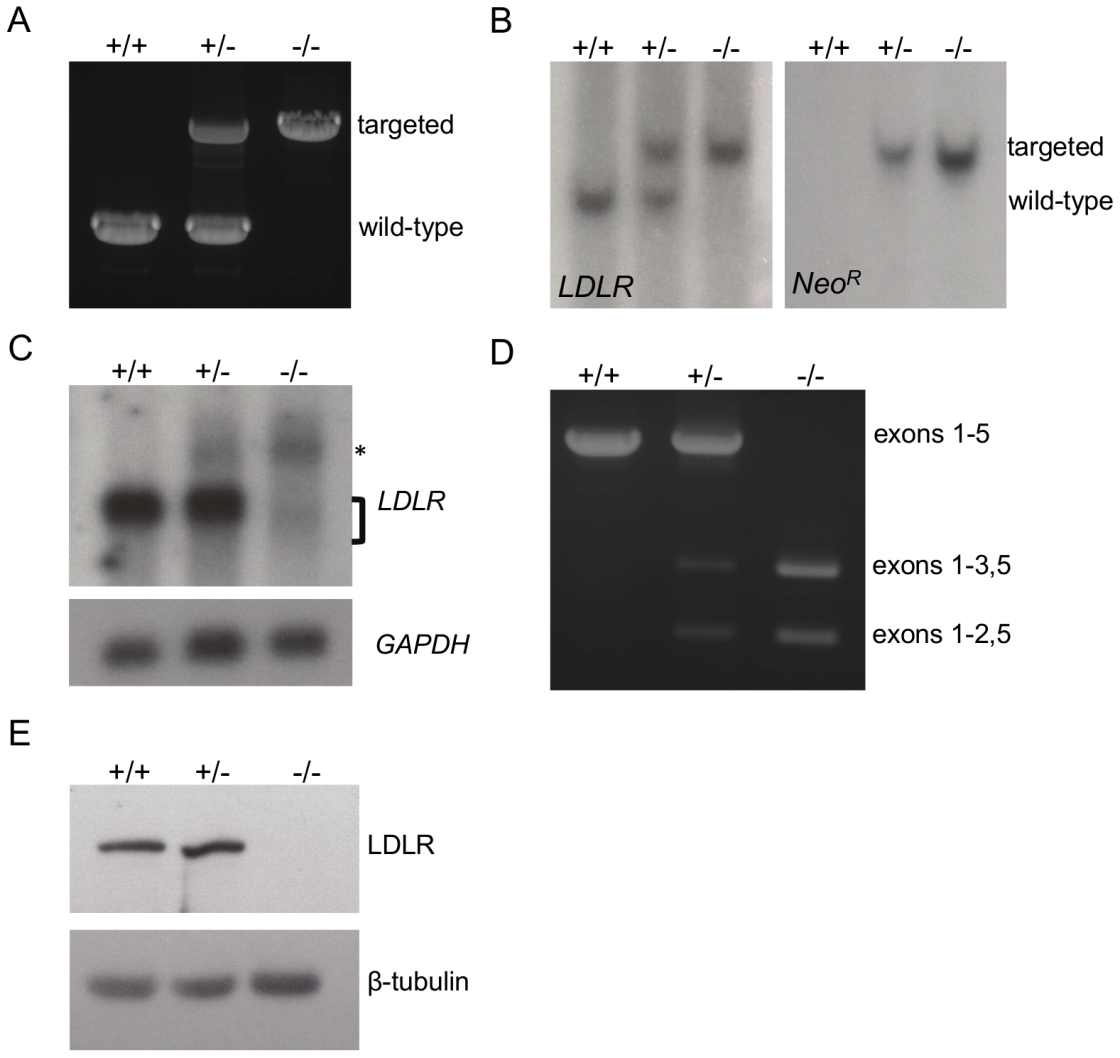
Molecular and biochemical characterization of *LDLR+/+*, *LDLR+/−*, and *LDLR−/−* pigs. A. Genotyping by PCR. Expected sizes are 1.5-type *LDLR* and 3.2 kb for targeted *LDLR*. B. Southern blot of genomic DNA. (Left) *XmnI* digested genomic DNA was hybridized with a probe that detects porcine *LDLR* downstream of the targeting vector boundary. The *LDLR*-targeted allele produced an approximately 7.8 kb band, and the wild-type band is approximately 6.0 kb. (Right) The same DNA was hybridized with a probe that detects the *Neo^R^* cassette, yielding only the targeted 7.8 kb band. C. Northern blot of *LDLR* and *GAPDH* mRNA. Full-length *LDLR* and *GAPDH* mRNAs are 5.1 and 1.5 kb, respectively. The asterisk represents a minor mRNA species consisting of full-length *LDLR* mRNA plus the *Neo^R^* cassette. The bracket indicates two minor mRNA species that are likely the result of nonsense-mediated mRNA altered splicing. D. Representative RT-PCR. Using PCR primers that amplify from exon 1 to exon 5, the targeted *LDLR* allele produces no normal mRNA, but does produce mRNA species with deletions of exon 4 or exons 3 and 4. This is seen in both the *LDLR+/−* and *LDLR−/−* pigs. This result was confirmed by DNA sequencing. E. Representative western blot of LDLR and β-tubulin. LDLR is ∼150 kDa and β-tubulin is 51 kDa.

Figure 2A,B shows representative genotyping results via PCR and Southern blot. As mentioned previously, we disrupted exon 4 with a *Neo^R^* cassette and inserted a premature termination codon. The most likely consequence of this mutation is the induction of nonsense-mediated mRNA decay [41]. However, should a protein be translated, it would be truncated in the ligand-binding domain, lack the transmembrane-spanning segment, and be non-functional [17]. An additional possibility could be the skipping of exon 4 via nonsense-associated altered splicing [42]. This, too, would result in a protein with no ability to bind LDL. Northern blot analysis of liver mRNA suggests that the targeted allele produces no normal *LDLR* transcripts (Figure 2C), and RT-PCR reveals the presence of truncated mRNAs resulting from transcripts lacking exon 4 as well as exons 3 and 4 (Figure 2D), each a scenario which should result in a frameshift mutation. DNA sequencing confirmed these results (data not shown). Finally, we evaluated all three genotypes for the presence of LDL receptor protein in the liver, which is the primary site of *LDLR* expression. Western blots confirmed that *LDLR−/−* pigs produce no normal LDL receptor protein (Figure 2E).

### Lipid chemistry analysis

Plasma cholesterol levels were measured in *LDLR+/+*, *LDLR+/−*, and *LDLR−/−* piglets immediately at birth, before piglets could suckle. This allows an initial assessment before lipid levels are affected by the sow's cholesterol- and fat-rich colostrum and milk. As expected, cholesterol levels were significantly higher in *LDLR*-targeted pigs than in their wild-type littermates. *LDLR+/−* piglets exhibited a moderate, but statistically significant total cholesterol elevation (similar to what was seen in *LDLR+/−* male clones described above), while *LDLR−/−* pigs had dramatically elevated levels (Table 2). A similar elevation is seen in LDL and very low-density lipoprotein (VLDL). High-density lipoprotein (HDL) was decreased in *LDLR−/−* pigs, though not in *LDLR+/−* pigs.

**Table 2:** Summary of lipid profiles from *LDLR+/+*, *LDLR+/−*, and *LDLR−/−* pigs at birth and after 26 weeks on a standard diet.

	Birth			26 weeks		
	*LDLR+/+* (5)	*LDLR+/−* (21)	*LDLR−/−* (10)	*LDLR+/+* (5)	*LDLR+/−* (17)	*LDLR−/−* (7)
**TC**	64.2±7.0	88.3±16.9*	490.8±19.0**	67.0±6.6	98.3±3.1*	567.4±34.3**
**LDL-C**	37.8±1.6	66.0±2.9*	451.2±18.7**	31.6±3.7	62.9±2.9**	434.7±26.7**
**VLDL-C**	4.8±0.7	4.3±0.3	30.7±2.2**	2.4±0.2	2.9±0.2	120.3±10.5**
**HDL-C**	21.4±2.0	18.4±0.8	8.9±0.8**	33.2±3.0	32.6±1.0	12.4±1.2**

All values are presented with SD. Differences between *LDLR+/+* and *LDLR+/−* are significant where indicated, ANOVA: *p<0.05, **p<0.01. Differences between *LDLR−/−* and the other two genotypes are significant where indicated, ANOVA: *p<0.01.

Cholesterol levels in pigs can vary widely while nursing (data not shown). At three to four weeks of age, pigs were weaned and placed on a standard diet consisting of no cholesterol and ∼2% fat. When measured again at 26 weeks of age, the cholesterol levels in these pigs were similar to what was seen pre-suckle. These cholesterol data are summarized in Table 2.

### Early atherosclerosis in *LDLR−/−* pigs

**Figure 3:**
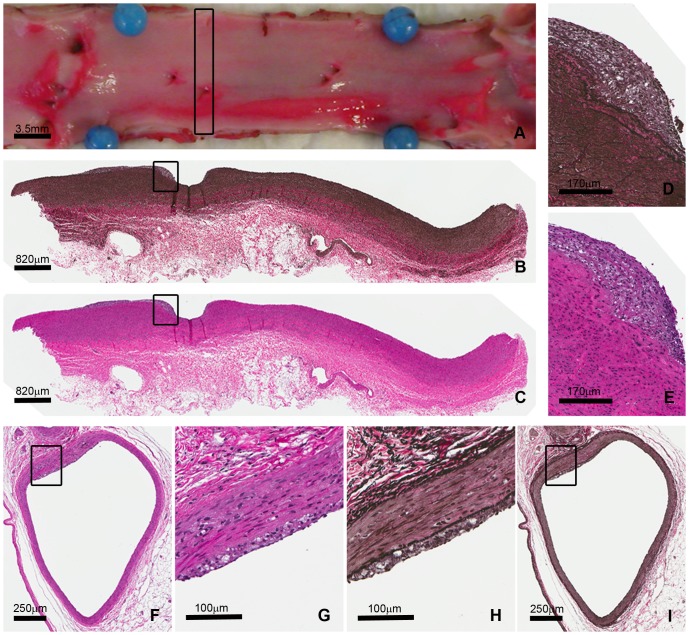
Abdominal aortic and coronary atherosclerosis in a 26-week-old *LDLR−/−* pig fed a standard diet. Measurements of percent surface area with raised lesion in the abdominal aorta were taken from en face photographs of the abdominal aorta and confirmed by Sudan IV staining (A). Aortic sections (rectangle) were stained with VVG and H&E (B,C), higher magnifications of atherosclerotic lesion (squares) are seen in D and E. A representative section (with corresponding higher magnification) from the circumflex artery showing atherosclerotic plaque that appears to have foam cells. (F,G–H&E H,I – VVG).

It is well established that elevated total and LDL cholesterol due to dietary manipulation will lead to the development of atherosclerosis in wild-type pigs. We asked whether *LDLR−/−* pigs would show the presence of atherosclerosis having been fed a standard pig diet with no added cholesterol or fat. We necropsied three *LDLR−/−* pigs and two *LDLR+/+* controls at 7 months of age and assessed the presence of atherosclerosis in the coronary arteries and the abdominal aorta. The *LDLR+/+* pigs had no visible atherosclerosis in the abdominal aorta, while the *LDLR−/−* pigs had 45% of the abdominal aorta covered with raised atherosclerotic lesions that were distributed fairly equally over proximal and distal portions. Atherosclerotic lesions had areas of probable foam cells and small areas of necrosis, hallmarks of human atherosclerosis (Figure 3A–E).

Coronary arteries from the *LDLR+/+* and *LDLR−/−* pigs were sectioned and stained with hemotoxylin and eosin (H&E) and Verhoeff-van Gieson (VVG) and evaluated for the presence of atherosclerosis. The *LDLR+/+* pigs had no visible atherosclerotic lesions in the right coronary artery (RCA), left anterior descending artery (LAD), or circumflex artery (CIRC). Small areas of intimal thickening were seen in the proximal portion of each. The *LDLR−/−* pigs however, had small atherosclerotic lesions, as well as intimal thickening in sections from the RCA, LAD, and CIRC. These were predominantly in the proximal portion of the arteries but there was some extension into the distal portions as well. A representative lesion is shown in Figure 3F–I. Lesions in the coronary arteries appear to have foam cells, a hallmark of human atherosclerosis.

### High fat, high cholesterol diet accelerates atherosclerosis

We next sought to increase the severity and accelerate the onset of atherosclerosis in the *LDLR*-targeted pigs by feeding a high fat, high cholesterol diet. For this study, we included only females and barrows (castrated males) since they are commonly used for atherosclerosis studies in industry settings. All pigs were raised on a standard diet until the age of 5 months, at which time they were transitioned to a diet containing ∼40% saturated fat and ∼1% cholesterol from natural sources including eggs and butter.

Cholesterol levels were measured following 90 and 180 days on the diet (Table 3). There was no difference in total cholesterol between females and barrows, so they have been combined within each genotype for this study. After 90 days of ingesting the high fat, high cholesterol diet, all three genotypes experienced significant increases in total cholesterol, which continued to increase at the 180-day time point.

**Table 3:** Summary of total cholesterol from *LDLR+/+*, *LDLR+/−*, and *LDLR−/−* pigs fed a high fat, high cholesterol diet for 90 and 180 days.

	*LDLR+/+*	*LDLR+/−*	*LDLR−/−*
**0 days**	78.6±8.6 (11)	105.3±18.6* (18)	619.1±81.7** (17)
**90 days**	174.4±38.2 (7)	250.2±75.6* (10)	834.0±117** (12)
**180 days**	161.0±23.6 (3)	323.8±92.3** (5)	960.4±46.9** (5)

All values are presented with SD. Differences between *LDLR+/+* and *LDLR+/−* are significant where indicated, ANOVA: *p<0.05, **p<0.01. Differences between *LDLR−/−* and the other two genotypes are significant where indicated, ANOVA: **p<0.01.

**Figure 4:**
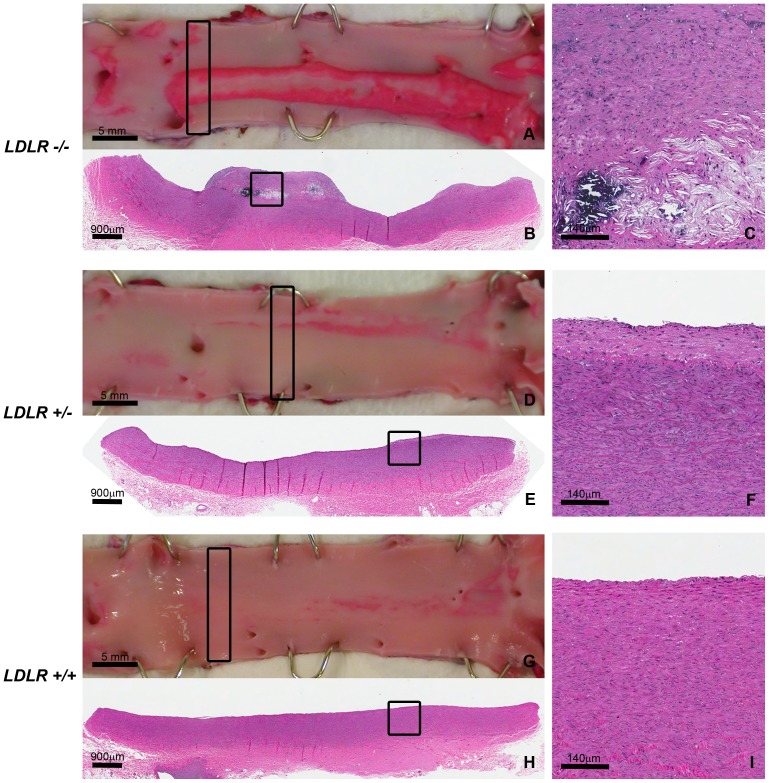
Abdominal aortic atherosclerosis in 11-month-old *LDLR−/−, LDLR +/−,* and *LDLR +/+* pigs fed a high fat, high cholesterol diet. Measurements of percent surface area with raised lesion in the abdominal aorta were taken from en face photographs of the abdominal aorta and confirmed by Sudan IV staining (A,D,G). Aortic sections (rectangles) were stained with H&E (B, E, H) and higher magnifications of atherosclerotic lesion (squares) are seen in C, F, and I. Representative sections from each are shown: *LDLR−/−* (A,B,C), *LDLR +/−* (D,E,F), and *LDLR +/+* (G,H,I). Atherosclerotic plaques from all the *LDLR−/−* pigs had cholesterol clefts and some lesions also exhibited calcification as shown in B and C.

The *LDLR−/−* pigs had an appreciable amount of atherosclerosis in the abdominal aorta. While the total abdominal aortic surface area was comparable in all three groups, *LDLR−/−* pigs had significantly higher surface area with raised lesions and percent aortic surface area covered with lesions (overall and in both the proximal and distal portions) than either the *LDLR+/−* or *LDLR+/+* pigs (Figure 4, Table 4). By morphometric methods, the intimal area and intimal area as percent of medial area were also significantly larger (overall and in both the proximal and distal portions) in the *LDLR−/−* pigs than either the *LDLR+/−* or *LDLR+/+* pigs (Table 4). All aortic atherosclerosis lesions from the *LDLR−/−* pigs had cholesterol clefts present and many had calcification and apparent foam cells (Figure 4).

**Table 4:** Abdominal Aortic Atherosclerosis from 11-month old *LDLR+/+, LDLR+/−*, and *LDLR−/−* pigs fed a high fat, high cholesterol diet.

	*LDLR +/+* (4)	*LDLR+/−* (6)	*LDLR−/−* (4)
**En Face Measurements:**			
**Abdominal Aorta**			
Total aortic surface area (cm^2^)	8.7±0.8	7.5±0.9	7.8±1.2
Area with raised lesion (cm^2^)	0.5±0.3	0.8±0.8	3.8±1.6**
% aortic surface with raised lesions	5.8±4.4	10.1±8.8	48.5±19.8**
**Proximal Half Abdominal Aorta**			
Total aortic surface area (cm^2^)	4.3±0.4	3.8±0.4	3.9±0.6
Area with raised lesion (cm^2^)	0.2±0.2	0.3±0.4	1.6±0.6**
% aortic surface with raised lesions	4.6±4.8	7.8±8.3	40.5±14.5**
**Distal Half Abdominal Aorta**			
Total aortic surface area (cm^2^)	4.4±0.4	3.7±0.5	3.9±0.6
Area with raised lesion (cm^2^)	0.3±0.2	0.5±0.4	2.2±1.1**
% aortic surface with raised lesions	7.0±5.2	12.3±9.8	56.6±26.7**
**Morphometry:**			
**Abdominal Aorta**			
Medial area (mm^2^)	11.9±0.6	12.3±1.4	12.4±1.4
Intimal area (mm^2^)	0.2±0.1	0.3±0.2	4.4±2.1**
Intimal area as % medial area	1.9±1.0	2.7±1.7	35.4±14.2**
**Proximal Half Abdominal Aorta**			
Medial area (mm^2^)	11.4±0.4	11.9±1.1	12.8±1.9
Intimal area (mm^2^)	0.1±0.0	0.2±0.2	3.6±1.1**
Intimal area as % medial area	1.2±0.4	1.9±2.0	27.4±5.1**
**Distal Half Abdominal Aorta**			
Medial area (mm^2^)	12.4±0.9	12.7±2.2	11.9±1.3
Intimal area (mm^2^)	0.4±0.2	0.5±0.2	5.4±3.2**
Intimal area as % medial area	2.7±1.7	3.6±1.7	43.8±23.4**

All values are presented with SD. Differences between *LDLR−/−* and the other two genotypes are significant where indicated, ANOVA: **p<0.01.

**Figure 5:**
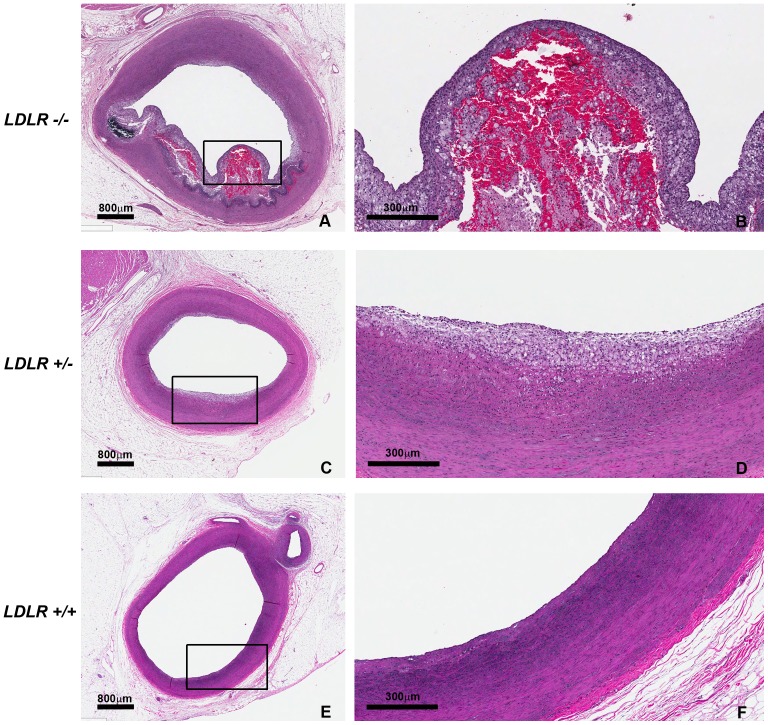
Coronary artery atherosclerosis in 11-month-old *LDLR−/−, LDLR +/−,* and *LDLR +/+* pigs fed a high fat, high cholesterol diet. Representative H&E stained sections from each group are shown: *LDLR−/−* (A,B), *LDLR +/−* (C,D), and *LDLR +/+* (E,F). Complex lesions were seen in the *LDLR−/−* pigs (A,B) that exhibited hemorrhage, possible areas of calcification, fibrous caps and foam cells.

After 180 days of the high fat, high cholesterol diet, coronary arteries from the 11-month old *LDLR+/+, LDLR+/−*, and *LDLR−/−* pigs were sectioned and stained with H&E and VVG and evaluated for the presence of atherosclerosis. The *LDLR+/+* pigs had only minimal amounts of intimal thickening in the RCA, LAD, and CIRC, while *LDLR+/−* pigs had small areas of atherosclerosis in addition to areas of intimal thickening. The *LDLR−/−* pigs however, had atherosclerotic lesions, as well as intimal thickening in sections from the RCA, LAD, and CIRC. Some lesions were complex with fibrous caps, hemorrhage, calcification, and foam cells (Figure 5).

By morphometric methods, the coronary arteries from *LDLR−/−* pigs had overall significantly larger intimal area, percent stenosis, and intimal area as percent medial area than the *LDLR+/+* or *LDLR+/−* coronary arteries. This difference was predominantly in the distal portion of the arteries with significantly larger percent stenosis and intimal area as percent medial area in the distal portion of coronary arteries from the *LDLR−/−* pigs (Table 5).

**Table 5:** Coronary Artery Atherosclerosis Morphometry from 11-month old *LDLR+/+*, *LDLR+/−*, and *LDLR−/−* pigs fed a high fat, high cholesterol diet.

	*LDLR +/+* (4)	*LDLR+/−* (6)		*LDLR−/−* (4)
**Coronary Arteries**				
Medial area (mm^2^)	0.7±0.2		0.9±0.2	1.0±0.2
Intimal area (mm^2^)	0.1±0.0		0.1±0.0	0.2±0.1 **
% stenosis	2.6±0.5		2.7±0.5	5.6±1.3 **
Intimal area as % medial area	10.2±2.6		9.8±1.7	15.2±2.7 *
**Proximal Coronary Arteries**				
Medial area (mm^2^)	0.9±0.3		1.1±0.3	1.4±0.3
Intimal area (mm^2^)	0.1±0.0		0.1±0.0	0.4±0.2 **
% stenosis	2.6±1.1		3.1±0.6	6.9±2.5 **
Intimal area as % medial area	11.5±3.8		11.1±2.0	18.1±4.6 *
**Distal Coronary Arteries**				
Medial area (mm^2^)	0.4±0.1		0.5±0.3	0.5±0.1
Intimal area (mm^2^)	0.0±0.0		0.0±0.0	0.1±0.0
% stenosis	2.4±0.5		2.1±0.5	4.0±1.0 *
Intimal area as % medial area	8.6±1.0		7.3±1.9	11.5±0.4*

All values are presented with SD. Differences between *LDLR−/−* and the other two genotypes are significant where indicated, ANOVA: *p<0.05, **p<0.01.

## Discussion

We have produced new models of hypercholesterolemia and coronary and aortic atherosclerosis in the commonly studied Yucatan miniature pig breed by disrupting the endogenous *LDLR* gene. *LDLR+/−* pigs exhibit mild, yet consistent elevated cholesterol that is present at birth and persists when fed a zero-cholesterol, low fat diet. *LDLR−/−* animals are born with severe hypercholesterolemia and develop atherosclerotic lesions by 7 months of age. The phenotypes of the *LDLR+/−* and *LDLR−/−* pigs are accelerated when fed a high fat, high cholesterol diet.

Our results are consistent with human FH, a dominant disorder exhibiting a gene dosage effect in which *LDLR* homozygotes exhibit a more severe phenotype than *LDLR* heterozygotes. Previous studies in Rapacz spontaneous hypercholesterolemic pigs (*LDLR-R84C* homozygotes) suggested that FH is a recessive disease in pigs, because cholesterol levels in heterozygotes did not differ from wild-type controls. However, this is likely due to the high genetic variability seen in these animals, including the presence of various *ApoB* alleles and other genetic modifiers [32]. The more homogenous genetic background of the *LDLR*-targeted Yucatan miniature pigs allows the difference between *LDLR+/+* and *LDLR+/−* animals to be more readily observed.

*LDLR*-targeted Yucatan miniature pigs offer several advantages over existing large animal models of hypercholesterolemia and atherosclerosis. First, at 60–75 kg, mature Yucatan miniature pigs are one-third to one-half the size of domestic pigs (including the Rapacz FH pigs) making them more representative of average human size and easier for most researchers to conduct studies. This is important for studies directed at the development of new human devices and diagnostic equipment. Second, due to the “knock-out” nature of the gene disruption, a more severe, and thus more rapidly developing, atherosclerosis is possible in the homozygotes compared to the Rapacz or PCSK9-D374Y transgenic pigs. This will decrease the duration and costs of studies. Furthermore, since the heterozygote pigs have only one functional *LDLR* allele, they are susceptible to accelerated, diet-induced hypercholesterolemia and atherosclerosis (compared to wild-type animals). This makes them uniquely suited for the development of therapeutic approaches that directly, or indirectly, induce LDL receptor expression and the evaluation of a treatment's ability to impede or even reverse the progression of atherosclerosis. This may not be possible with the constitutive transgene expression in the PCSK9-D374Y pig. Finally, because of the homogeneous genetic background of the miniature pig line, the variability in phenotype and response to interventions seen in current models should also be reduced.

This model also has some limitations, with perhaps the greatest being its size and the challenges that come with it. A large animal model requires specialized housing, trained staff, unique study facilities, and in some cases, specially formulated diets. Most current animal models of hypercholesterolemia and atherosclerosis require a highly modified diet often consisting of 1–4% pure cholesterol and sodium cholate, which are not typical of human diets and with the latter being possibly toxic and counterproductive [43]. Furthermore, these diets are cost-prohibitive for many researchers, with daily feed costs being ∼40 times more expensive than normal swine diets. We used a diet that is more representative of human consumption containing natural sources of saturated fat and cholesterol, which is significantly cheaper (2-3X normal swine diets). A pig's size is not ideal for early-stage drug development, due to the amount of test compound required. Accordingly, pigs may be better suited to bridge the large gap that exists between small animal models and human clinical trials. For example, several recent studies have shown promising results in the current small animal models of atherosclerosis, only to fail in phase 3 studies [44]. If the porcine model were to offer more predictive efficacy, the benefits would far outweigh the costs. Another limitation is that pigs, like mice, lack cholesteryl ester transfer protein (CETP). Inhibition of human CETP to increase circulating HDL has been an area of intense study recently, however to date, no drugs in this class have progressed beyond clinical trials [45].

Several issues regarding the *LDLR*-targeted Yucatan miniature pigs remain to be addressed. We describe the presence of advanced atherosclerotic lesions in eleven-month-old *LDLR−/−* pigs fed a high fat, high cholesterol diet, but we did not observe plaque rupture or thrombosis. A longer study is underway to better establish the full spectrum and timeline of phenotypic development, though the results presented here are encouraging. The present study also does not address the impact of gender and puberty on phenotype. Our standard diet study only looked at pigs up to 6–7 months, which is the point at which pigs undergo puberty. We saw no discernable differences between males and females up to that point. The high fat, high cholesterol diet study only looked at castrated males and females, and again, no differences in atherosclerosis severity were seen. Finally, it is obvious that an animal model with improved predictive efficacy is needed. While that will take more time to demonstrate, we are currently seeking to validate that drugs that have been successful in humans are equally effective in the porcine model. Perhaps more importantly, we would also like to know whether *LDLR*-targeted pigs would have predicted the failure in humans of drugs that were initially shown to be efficacious in mice.

The *LDLR*-targeted Yucatan miniature pigs provide a versatile new model system for pursuing research into new devices, diagnostic tools, and therapeutics for human cardiovascular disease. This study further validates genetically engineered pigs as useful biomedical models of human disease.

## Supporting Information

Figure S1 **Genotyping results from *LDLR*-targeted pig fetal fibroblasts.** A. Example of PCR results. Primers amplified a 1.5 kb product from the wild-type allele and 3.2 kb product from the *LDLR*-targeted allele. Lanes 1–5 are examples of PCR-positive cell clones. Lane 6 is a wild-type cell clone. B. Southern blot of whole genome amplified DNA. (Left) *XmnI* digested genomic DNA was hybridized with a probe that detects porcine *LDLR* downstream of the targeting vector boundary. The *LDLR*-targeted allele produced an approximately 7.8 kb band, and the wild-type band is approximately 6.0 kb. (Right) The same DNA was hybridized with a probe that detects the *Neo^R^* cassette, yielding only the targeted 7.8 kb band. Lane 3 is an example of a properly targeted cell line. Lane 5 is a wild-type control.
(PDF)

Table S1 **List of PCR and Sequencing Primers.**
(PDF)

## References

[1] Rogers CS, Hao Y, Rokhlina T, Samuel M, Stoltz DA, et al (2008) Production of CFTR-null and CFTR-DeltaF508 heterozygous pigs by adeno-associated virus-mediated gene targeting and somatic cell nuclear transfer. J Clin Invest 118: 1571–1577. 18324337 10.1172/JCI34773 PMC2265103

[2] Rogers CS, Stoltz DA, Meyerholz DK, Ostedgaard LS, Rokhlina T, et al (2008) Disruption of the CFTR gene produces a model of cystic fibrosis in newborn pigs. Science 321: 1837–1841. 18818360 10.1126/science.1163600 PMC2570747

[3] Stoltz DA, Meyerholz DK, Pezzulo AA, Ramachandran S, Rogan MP, et al (2010) Cystic fibrosis pigs develop lung disease and exhibit defective bacterial eradication at birth. Sci Transl Med 2: 29ra31. 10.1126/scitranslmed.3000928 PMC2889616 20427821

[4] Ostedgaard LS, Meyerholz DK, Chen JH, Pezzulo AA, Karp PH, et al (2011) The DeltaF508 mutation causes CFTR misprocessing and cystic fibrosis-like disease in pigs. Sci Transl Med 3: 74ra24. 10.1126/scitranslmed.3001868 PMC3119077 21411740

[5] Rogan MP, Reznikov LR, Pezzulo AA, Gansemer ND, Samuel M, et al (2010) Pigs and humans with cystic fibrosis have reduced insulin-like growth factor 1 (IGF1) levels at birth. Proc Natl Acad Sci U S A 107: 20571–20575. 21059918 10.1073/pnas.1015281107 PMC2996661

[6] Pezzulo AA, Tang XX, Hoegger MJ, Alaiwa MH, Ramachandran S, et al (2012) Reduced airway surface pH impairs bacterial killing in the porcine cystic fibrosis lung. Nature 487: 109–113. 22763554 10.1038/nature11130 PMC3390761

[7] Johnson GJ, Griggs TR, Badimon L (1999) The utility of animal models in the preclinical study of interventions to prevent human coronary artery restenosis: analysis and recommendations. On behalf of the Subcommittee on Animal, Cellular and Molecular Models of Thrombosis and Haemostasis of the Scientific and Standardization Committee of the International Society on Thrombosis and Haemostasis. Thromb Haemost 81: 835–843. 10365761

[8] Lusis AJ, Mar R, Pajukanta P (2004) Genetics of atherosclerosis. Annu Rev Genomics Hum Genet 5: 189–218. 15485348 10.1146/annurev.genom.5.061903.175930

[9] Sanz J, Moreno PR, Fuster V (2013) The year in atherothrombosis. J Am Coll Cardiol 62: 1131–1143. 23916939 10.1016/j.jacc.2013.06.045

[10] Libby P, Lichtman AH, Hansson GK (2013) Immune effector mechanisms implicated in atherosclerosis: from mice to humans. Immunity 38: 1092–1104. 23809160 10.1016/j.immuni.2013.06.009 PMC3764500

[11] Tabas I, Williams KJ, Boren J (2007) Subendothelial lipoprotein retention as the initiating process in atherosclerosis: update and therapeutic implications. Circulation 116: 1832–1844. 17938300 10.1161/CIRCULATIONAHA.106.676890

[12] Little WC, Constantinescu M, Applegate RJ, Kutcher MA, Burrows MT, et al (1988) Can coronary angiography predict the site of a subsequent myocardial infarction in patients with mild-to-moderate coronary artery disease? Circulation 78: 1157–1166. 3180375 10.1161/01.cir.78.5.1157

[13] Stone GW, Maehara A, Lansky AJ, de Bruyne B, Cristea E, et al (2011) A prospective natural-history study of coronary atherosclerosis. N Engl J Med 364: 226–235. 21247313 10.1056/NEJMoa1002358

[14] Nichols TC (2013) Bad cholesterol breaking really bad. Blood 122: 3551–3553. 24263954 10.1182/blood-2013-09-527697 PMC3837505

[15] Rader DJ, Cohen J, Hobbs HH (2003) Monogenic hypercholesterolemia: new insights in pathogenesis and treatment. J Clin Invest 111: 1795–1803. 12813012 10.1172/JCI18925 PMC161432

[16] Daugherty A (2002) Mouse models of atherosclerosis. Am J Med Sci 323: 3–10. 11814139 10.1097/00000441-200201000-00002

[17] Ishibashi S, Brown MS, Goldstein JL, Gerard RD, Hammer RE, et al (1993) Hypercholesterolemia in low density lipoprotein receptor knockout mice and its reversal by adenovirus-mediated gene delivery. J Clin Invest 92: 883–893. 8349823 10.1172/JCI116663 PMC294927

[18] Plump AS, Smith JD, Hayek T, Aalto-Setala K, Walsh A, et al (1992) Severe hypercholesterolemia and atherosclerosis in apolipoprotein E-deficient mice created by homologous recombination in ES cells. Cell 71: 343–353. 1423598 10.1016/0092-8674(92)90362-g

[19] Purcell-Huynh DA, Farese RV Jr, Johnson DF, Flynn LM, Pierotti V, et al (1995) Transgenic mice expressing high levels of human apolipoprotein B develop severe atherosclerotic lesions in response to a high-fat diet. J Clin Invest 95: 2246–2257. 7738190 10.1172/JCI117915 PMC295837

[20] Bentzon JF, Falk E (2010) Atherosclerotic lesions in mouse and man: is it the same disease? Curr Opin Lipidol 21: 434–440. 20683327 10.1097/MOL.0b013e32833ded6a

[21] Benvenuti LA, Onishi RY, Gutierrez PS, de Lourdes Higuchi M (2005) Different patterns of atherosclerotic remodeling in the thoracic and abdominal aorta. Clinics (Sao Paulo) 60: 355–360. 16254670 10.1590/s1807-59322005000500002

[22] Kobayashi T, Ito T, Shiomi M (2011) Roles of the WHHL rabbit in translational research on hypercholesterolemia and cardiovascular diseases. J Biomed Biotechnol 2011: 406473. 21541231 10.1155/2011/406473 PMC3085394

[23] Gerrity RG, Natarajan R, Nadler JL, Kimsey T (2001) Diabetes-induced accelerated atherosclerosis in swine. Diabetes 50: 1654–1665. 11423488 10.2337/diabetes.50.7.1654

[24] Schwartz RS, Edelman E, Virmani R, Carter A, Granada JF, et al (2008) Drug-eluting stents in preclinical studies: updated consensus recommendations for preclinical evaluation. Circ Cardiovasc Interv 1: 143–153. 20031669 10.1161/CIRCINTERVENTIONS.108.789974 PMC2935144

[25] Onuma Y, Serruys PW, Perkins LE, Okamura T, Gonzalo N, et al (2010) Intracoronary optical coherence tomography and histology at 1 month and 2, 3, and 4 years after implantation of everolimus-eluting bioresorbable vascular scaffolds in a porcine coronary artery model: an attempt to decipher the human optical coherence tomography images in the ABSORB trial. Circulation 122: 2288–2300. 20975003 10.1161/CIRCULATIONAHA.109.921528

[26] Gerrity RG, Goss JA, Soby L (1985) Control of monocyte recruitment by chemotactic factor(s) in lesion-prone areas of swine aorta. Arteriosclerosis 5: 55–66. 3966908 10.1161/01.atv.5.1.55

[27] Thomas WA, Lee KT, Kim DN (1985) Cell population kinetics in atherogenesis. Cell births and losses in intimal cell mass-derived lesions in the abdominal aorta of swine. Ann N Y Acad Sci 454: 305–315. 3865613 10.1111/j.1749-6632.1985.tb11870.x

[28] Mohiaddin RH, Burman ED, Prasad SK, Varghese A, Tan RS, et al (2004) Glagov remodeling of the atherosclerotic aorta demonstrated by cardiovascular magnetic resonance: the CORDA asymptomatic subject plaque assessment research (CASPAR) project. J Cardiovasc Magn Reson 6: 517–525. 15137336 10.1081/jcmr-120030576

[29] Dixon JL, Stoops JD, Parker JL, Laughlin MH, Weisman GA, et al (1999) Dyslipidemia and vascular dysfunction in diabetic pigs fed an atherogenic diet. Arterioscler Thromb Vasc Biol 19: 2981–2992. 10591679 10.1161/01.atv.19.12.2981

[30] Hasler-Rapacz J, Ellegren H, Fridolfsson AK, Kirkpatrick B, Kirk S, et al (1998) Identification of a mutation in the low density lipoprotein receptor gene associated with recessive familial hypercholesterolemia in swine. Am J Med Genet 76: 379–386. 9556295

[31] Rapacz J, Hasler-Rapacz J, Taylor KM, Checovich WJ, Attie AD (1986) Lipoprotein mutations in pigs are associated with elevated plasma cholesterol and atherosclerosis. Science 234: 1573–1577. 3787263 10.1126/science.3787263

[32] Grunwald KA, Schueler K, Uelmen PJ, Lipton BA, Kaiser M, et al (1999) Identification of a novel Arg—>Cys mutation in the LDL receptor that contributes to spontaneous hypercholesterolemia in pigs. J Lipid Res 40: 475–485. 10064736

[33] Prescott MF, McBride CH, Hasler-Rapacz J, Von Linden J, Rapacz J (1991) Development of complex atherosclerotic lesions in pigs with inherited hyper-LDL cholesterolemia bearing mutant alleles for apolipoprotein B. Am J Pathol 139: 139–147. 1853929 PMC1886122

[34] Thim T, Hagensen MK, Drouet L, Bal Dit Sollier C, Bonneau M, et al (2010) Familial hypercholesterolaemic downsized pig with human-like coronary atherosclerosis: a model for preclinical studies. EuroIntervention 6: 261–268. 20562079 10.4244/EIJV6I2A42

[35] Al-Mashhadi RH, Sorensen CB, Kragh PM, Christoffersen C, Mortensen MB, et al (2013) Familial hypercholesterolemia and atherosclerosis in cloned minipigs created by DNA transposition of a human PCSK9 gain-of-function mutant. Sci Transl Med 5: 166ra161. 10.1126/scitranslmed.3004853 23283366

[36] Lai L, Prather RS (2003) Production of cloned pigs by using somatic cells as donors. Cloning Stem Cells 5: 233–241. 14733743 10.1089/153623003772032754

[37] Pomp D, Good BA, Geisert RD, Corbin CJ, Conley AJ (1995) Sex identification in mammals with polymerase chain reaction and its use to examine sex effects on diameter of day-10 or -11 pig embryos. J Anim Sci 73: 1408–1415. 7665371 10.2527/1995.7351408x

[38] Walker SC, Shin T, Zaunbrecher GM, Romano JE, Johnson GA, et al (2002) A highly efficient method for porcine cloning by nuclear transfer using in vitro-matured oocytes. Cloning Stem Cells 4: 105–112. 12171703 10.1089/153623002320253283

[39] Hobbs HH, Russell DW, Brown MS, Goldstein JL (1990) The LDL receptor locus in familial hypercholesterolemia: mutational analysis of a membrane protein. Annu Rev Genet 24: 133–170. 2088165 10.1146/annurev.ge.24.120190.001025

[40] Hickey RD, Lillegard JB, Fisher JE, McKenzie TJ, Hofherr SE, et al (2011) Efficient production of Fah-null heterozygote pigs by chimeric adeno-associated virus-mediated gene knockout and somatic cell nuclear transfer. Hepatology 54: 1351–1359. 21674562 10.1002/hep.24490 PMC3184202

[41] Wen J, Brogna S (2008) Nonsense-mediated mRNA decay. Biochem Soc Trans 36: 514–516. 18481993 10.1042/BST0360514

[42] Wang J, Chang YF, Hamilton JI, Wilkinson MF (2002) Nonsense-associated altered splicing: a frame-dependent response distinct from nonsense-mediated decay. Mol Cell 10: 951–957. 12419238 10.1016/s1097-2765(02)00635-4

[43] Lichtman AH, Clinton SK, Iiyama K, Connelly PW, Libby P, et al (1999) Hyperlipidemia and atherosclerotic lesion development in LDL receptor-deficient mice fed defined semipurified diets with and without cholate. Arterioscler Thromb Vasc Biol 19: 1938–1944. 10446074 10.1161/01.atv.19.8.1938

[44] Agarwala A, Billheimer J, Rader DJ (2013) Mighty minipig in fight against cardiovascular disease. Sci Transl Med 5: 166fs161. 10.1126/scitranslmed.3005369 23283365

[45] Mohammadpour AH, Akhlaghi F (2013) Future of cholesteryl ester transfer protein (CETP) inhibitors: a pharmacological perspective. Clin Pharmacokinet 52: 615–626. 23658137 10.1007/s40262-013-0071-8 PMC3720705

