# Supplementary material for: Targeted Disruption of LDLR Causes Hypercholesterolemia and Atherosclerosis in Yucatan Miniature Pigs
Source: PLoS One. 2014 Apr 1;9(4):e93457. doi: 10.1371/journal.pone.0093457 (PMC3972179; doi:10.1371/journal.pone.0093457)
Supplement: Figure S1 — Genotyping results from LDLR-targeted pig fetal fibroblasts. A. Example of PCR results. Primers amplified a 1.5 kb product from the wild-type allele and 3.2 kb product from the LDLR-targeted allele. Lanes 1–5 are examples of PCR-positive cell clones. Lane 6 is a wild-type cell clone. B. Southern blot of whole genome amplified DNA. (Left) XmnI digested genomic DNA was hybridized with a probe that detects porcine LDLR downstream of the targeting vector boundary. The LDLR-targeted allele produced an approximately 7.8 kb band, and the wild-type band is approximately 6.0 kb. (Right) The same DNA was hybridized with a probe that detects the NeoR cassette, yielding only the targeted 7.8 kb band. Lane 3 is an example of a properly targeted cell line. Lane 5 is a wild-type control. (PDF) [file pone.0093457.s001.pdf]

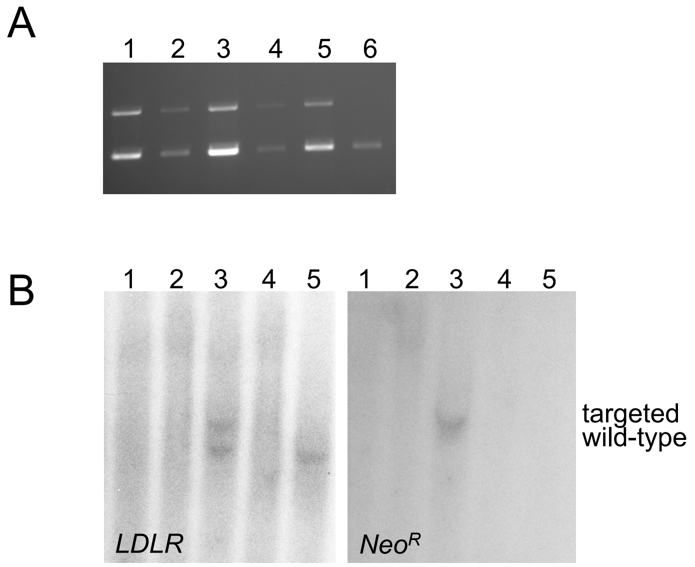

Figure S1. Genotyping results from *LDLR*-targeted pig fetal fibroblasts. A. Example of PCR results. Primers amplified a 1.5 kb product from the wild-type allele and 3.2 kb product from the *LDLR*-targeted allele. Lanes 1-5 are examples of PCR-positive cell clones. Lane 6 is a wild-type cell clone. B. Southern blot of whole genome amplified DNA. (Left) *XmnI* digested genomic DNA was hybridized with a probe that detects porcine *LDLR* downstream of the targeting vector boundary. The *LDLR*-targeted allele produced an approximately 7.8 kb band, and the wild-type band is approximately 6.0 kb. (Right) The same DNA was hybridized with a probe that detects the Neo^R cassette, yielding only the targeted 7.8 kb band. Lane 3 is an example of a properly targeted cell line. Lane 5 is a wild-type control.
