# Supplementary material for: Targeted Disruption of LDLR Causes Hypercholesterolemia and Atherosclerosis in Yucatan Miniature Pigs
Source: PLoS One. 2014 Apr 1;9(4):e93457. doi: 10.1371/journal.pone.0093457 (PMC3972179; doi:10.1371/journal.pone.0093457)
Supplement: Table S1 — List of PCR and Sequencing Primers. (PDF) [file pone.0093457.s002.pdf]

Table S1. List of PCR and Sequencing Primers

**PCR primers**	**Sequence (5' to 3')**
2F2	GGAAATGCATCTCCTACAAGT
6R2	GCAGTCCCTGACTGAGGTG
LDLR5'armF (*Xho*I)	gatcca*ctcgag*GTATTGATGCATTCTGCTGTG
LDLR5'armR (*EcoR*V)	gcatat*gatatc*ctaGACAAACTTTGGGGCGATGC
LDLR3'armF (*Hind*III)	tagcct*aagctt*GACTCGGACCGGGACTGCC
LDLR3'armR (*Hind*III)	tagcct*aagctt*GCACAGTGAGGCTAAGTCC
AAV-LDLR-F (*Not*I)	gatc*gcggccgc*CCATCTTGACCAAAAGCTGC
AAV-LDLR-R (*Not*I)	gatc*gcggccgc*CTGAGCCACCATGGGAACT
LDLR Ex4aF1	CAAGACGTGCTCCCAAGATG
LDLR Int4R1	AGCCATAGTAGGTCCTGATG
LDLR North 4F	ATGAAGTCCACGGGCTGGGTC
LDLR Exon 5R1	AGCACTGGAACTCGTCAGG
LDLR XmnI probe1F	GTAATTGAAGTAGTCAGCTTATC
LDLR XmnI probe1R	ATAAGTGAGTGCAACTCTGA
PGK-NeoF	CCAGTGTGCTGGAATTCGG
NeoR-R	CTCGTCAAGAAGGCGATAGAA
LDLR North8F	GACAGGGAATATGACTGCAAG
LDLR North9R	ACATCGATGCTGGAGATGGAG
pGAPDH North1F	ATGGTGAAGGTCGGAGTGAAC
pGAPDH North2R	CTCTTACTCCTTGGAGGCCAT
**Sequencing primers**	
LDLR 7F	AGTCAGAGCAAAGCAGTAATC
LDLR 8R	GTCACTTGGACGTGGCTGA
LDLR Ex3F1	GTGTCAACCGCTGCATTCC
LDLR 4F	ACAAGAAATTGAATGTGGATCC
LDLR Ex4aF1	CAAGACGTGCTCCCAAGATG
LDLR 4R	CTTTCGACCTGCAGCCAATA
ScreenF (NeoR)	AGACGTGCTACTTCCATTTGTCAC
LDLR 1R	GGAGCATGCGCTTTAGCAG
PGK 41R	CTGCTAAAGCGCATGCTCC
LDLR 5F	AGCCACAGCTCATCACTCC
GC6 F	TAGCTAGAGGCTGGAAGCAG

Underlined nucleotides indicate an introduced termination codon and italicized nucleotides indicate restriction enzyme sequences.
